# Effect of the similarity of gut microbiota composition between donor and recipient on graft function after living donor kidney transplantation

**DOI:** 10.1038/s41598-020-76072-8

**Published:** 2020-11-03

**Authors:** Ji Eun Kim, Hyo-Eun Kim, Hyunjeong Cho, Ji In Park, Min-Jung Kwak, Byung-Yong Kim, Seung Hee Yang, Jung Pyo Lee, Dong Ki Kim, Kwon Wook Joo, Yon Su Kim, Bong-Soo Kim, Hajeong Lee

**Affiliations:** 1 Department of Internal Medicine, Seoul National University Hospital, 103 Daehakro, Jongno-gu, Seoul, 03080 Republic of Korea; 2 Department of Internal Medicine, Korea University Guro Hospital, Seoul, Republic of Korea; 3 Biomedical Research Institute, Seoul National University Hospital, Seoul, Republic of Korea; 4 Department of Internal Medicine, Chungbuk National University Hospital, Cheongju, Republic of Korea; 5 Department of Internal Medicine, Kangwon National University Hospital, Chuncheon, Republic of Korea; 6 ChunLab, Inc, Seoul, Republic of Korea; 7 Kidney Research Institute, Seoul National University, Seoul, Republic of Korea; 8 Department of Internal Medicine, Seoul National University Boramae Hospital, Seoul, Republic of Korea; 9 Department of Life Science, Multidisciplinary Genome Institute, Hallym University, Chuncheon, Republic of Korea; 10 Department of Internal Medicine, Seoul National University College of Medicine, Seoul, Republic of Korea

**Keywords:** Nephrology, Microbiology

## Abstract

Graft outcomes of unrelated donor kidney transplant are comparable with those of related donor kidney transplant despite their genetic distance. This study aimed to identify whether the similarity of donor–recipient gut microbiota composition affects early transplant outcomes. Stool samples from 67 pairs of kidney transplant recipients and donors were collected. Gut microbiota differences between donors and recipients were determined using weighted UniFrac distance. Among the donor–recipient pairs, 30 (44.8%) pairs were related, while 37 (55.2%) were unrelated. The unrelated pairs, especially spousal pairs, had similar microbial composition, and they more frequently shared their meals than related pairs did. The weighted UniFrac distance showed an inverse correlation with the 6-month allograft function (*p* = 0.034); the correlation was significant in the unrelated pairs (*p* = 0.003). In the unrelated pairs, the microbial distance showed an excellent accuracy in predicting the estimated glomerular filtration rate of < 60 mL/min/1.73 m^2^ at 6-months post-transplantation and was better than human leukocyte antigen incompatibility and rejection. The incidence of infection within 6 months post-transplantation increased in the recipients having dissimilar microbiota with donors compared to the other recipients. Thus, pre-transplantation microbial similarity in unrelated donors and recipients may be associated with 6-month allograft function.

## Introduction

Owing to an increase in the number of patients with end-stage renal disease (ESRD), there is a growing demand for kidney transplantation, which is the most effective treatment for ESRD^1,2^. However, the waiting time for kidney transplantation is increasing annually due to the deficiency in the available donor pool^2^. Furthermore, the proportion of kidney transplantations from unrelated donors rather than related donors has been increasing recently^2^. Previously, unrelated donor transplantation was less common because of concerns about the genetic difference between the unrelated donors and recipients, and its negative effect on graft outcome. Yet, several studies have shown that transplantation outcomes with unrelated donors were not inferior to those with related donors^3–5^. Therefore, unrelated donors are considered safe, and this has expanded the donor sources. Comparable transplant outcomes between related and unrelated donors may be attributed to the development of potent immunosuppressants, improved immunologic monitoring systems, or meticulous supportive care, but the effects of environmental factors that compensate for genetic differences remain unknown.

One of the important environmental factors influencing diseases is the human microbiome^6^. It has been estimated that the size of the total number of genes in commensal microbial genomes is hundreds of times higher than the number of genes in the human genome^7^. Growing evidence indicates that microorganisms in the human body are related to various diseases, and some diseases have been associated with gut microbiome alteration, generally via the loss of microbial diversity, particularly, the depletion of specific bacteria^6^. Interestingly, these characteristics of microorganisms in an individual can change depending on the environment. Humans create their own “microbial cloud” by constantly shedding microbiota^8^. Thus, an intimate relationship and sharing common areas might result in exchanging microbes between individuals^9–11^. Furthermore, in a recent study, cohabitation was reported to be an important factor for immunological variation, suggesting the possible immunological effect of sharing microbes^12^. Additionally, treatment of some metabolic diseases by sharing microorganisms among certain healthy individuals is being investigated^6,13^.

Although the importance of sharing microbes and the similarity of microbiota among individuals has been reported, there are not many microbial studies in the field of kidney transplantation^14–16^. In particular, the effects of microbiota differences between donors and recipients on transplantation outcomes have not been considered to date. Therefore, this study was conducted to determine whether the similarity of microbiota between donors and recipients affects the transplant outcome.

## Results

### Clinical characteristics of transplant pairs and group classification

Sixty-seven donor–recipient transplant pairs were enrolled in this study. The recipients were 47.8 ± 13.0 years, and donors were 48.0 ± 11.0 years. Among the recipients, 56.7% were male, and among the donors, 59.7% were female. Among the 67 pairs, there were 30 pairs of relatives, 32 pairs of married couples, and 5 pairs of unrelated individuals.

All participants collected stool samples before transplantation, and longest collection period before transplant was 85 days. A total of 4,941,256 reads (average 37,135 ± 14,013 reads in donor samples and 36,615 ± 18,553 reads in recipient samples) were obtained from 16S rRNA gene sequencing results (Supplementary Table 1). Quantified microbial distances, represented by weighted UniFrac distance, ranged between 0.22 and 0.77, with an average value of 0.44 ± 0.13. Microbial distances were divided by tertiles and classified as ‘the most similar’, ‘similar,’ and ‘dissimilar’ groups, and then their baseline characteristics were compared (Table 1). Sex, the presence of diabetes mellitus (DM), and donor type of recipients were significantly different among the three distance-based groups. Among the donor types, 26/32 pairs (81.6%) of spousal donor transplants were included in ‘the most similar’ and ‘similar’ distance groups, whereas 15/30 pairs (50%) of the relative donors were included in the ‘dissimilar’ group. The dissimilar group received their allograft from a higher proportion of genetically related donors (62.5%) than the most similar (30.0%) or similar (39.1%) groups (*p* = 0.048). In contrast, the rate of ABO- and human leukocyte antigen (HLA)-incompatible transplants, and the number of HLA mismatches did not differ among the groups. In a survey about the frequency of sharing meals to estimate the strength of interaction and intimacy between the donor and recipient pairs, the average number of meals shared in a day was found to be higher in unrelated pairs (spouse and others) than in related pairs (*p* = 0.024; Supplementary Fig. 1).

**Table 1:** Baseline characteristics of recipients and donors according to the microbial distance groups.

Variables	Most similar (1st tertile)	Similar (2nd tertile)	Dissimilar (3rd tertile)	*P*
	N = 20	N = 23	N = 24	
**Recipient**				
Age (years)	49.5 ± 10.6	47.9 ± 13.6	46.4 ± 14.6	0.871
Male gender (%)	17 (85.0)	12 (52.2)	9 (37.5)	0.006
BMI (kg/m^2^)	23.9 ± 2.5	22.8 ± 4.0	22.3 ± 5.0	0.227
DM (%)	10 (50.0)	7 (30.4)	3 (12.5)	0.026
HTN (%)	17 (85.0)	17 (73.9)	16 (66.7)	0.378
Cause of end-stage renal disease				0.247
DM	8 (40.0)	5 (21.7)	3 (12.5)	
HTN	0 (0)	1 (4.3)	0 (0)	
Glomerulonephritis	6 (30.0)	8 (34.8)	7 (29.2)	
Polycystic kidney disease	3 (15.0)	2 (8.7)	2 (8.3)	
Unknown	3 (15.0)	7 (30.4)	12 (50.0)	
Dialysis duration (months)	7.4 ± 14.4	12.1 ± 16.4	13.3 ± 32.4	0.163
ABO incompatibility (%)	6 (30.0)	5 (21.7)	7 (29.2)	0.790
HLA incompatibility (%)	1 (5.0)	3 (13.0)	1 (12.5)	0.635
Number of HLA mismatch	3.6 ± 1.8	3.4 ± 1.8	2.5 ± 1.6	0.061
Donor type (%)				0.048
Related	6 (30.0)	9 (39.1)	15 (62.5)	
Unrelated (Spouse + others)	14 (70.0)	14 (60.9)	9 (37.5)	
Spouse	12 (60.0)	14 (60.9)	6 (25.0)	
Others	2 (10.0)	0 (0)	3 (12.5)	
**Donor**				
Age (years)	46.2 ± 10.7	51.6 ± 7.9	45.8 ± 13.2	0.228
Male gender (%)	7 (35.0)	9 (39.1)	11 (45.8)	0.759
BMI (kg/m^2^)	24.3 ± 3.3	24.6 ± 3.5	23.4 ± 2.9	0.541
Weighted UniFrac distance	0.29 ± 0.04	0.43 ± 0.04	0.59 ± 0.07	< 0.001

*BMI* body mass index, *DM* diabetes mellitus, *HTN* hypertension, *HLA* human leukocyte antigen.

**Figure 1:**
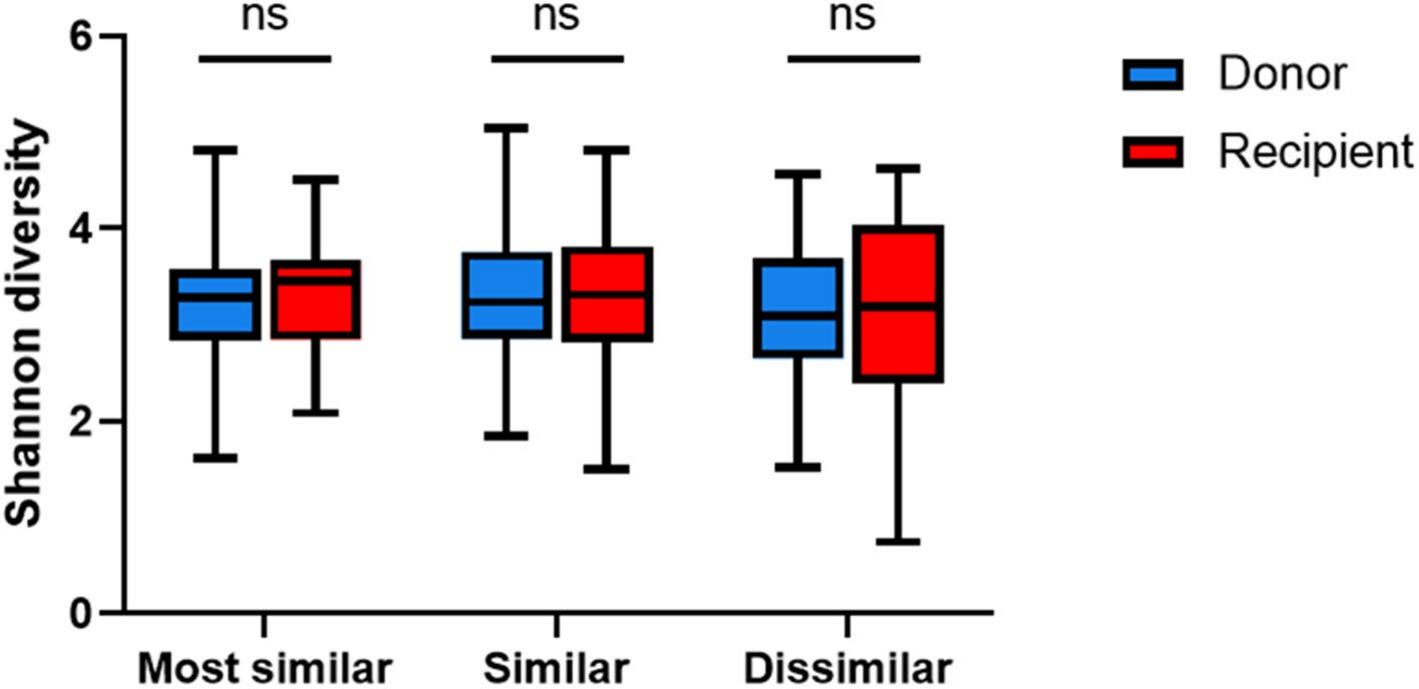
Shannon diversities of donors and recipients according to distance groups. Blue bars: donors, red bars: recipients.

### Comparison of diversity and microbiota according to the microbial distance group

The Shannon diversity index was not significantly different between donors and recipients in all groups (*p* = 0.512, 0.965 and 0.752 for most similar, similar, and dissimilar groups, respectively. Figure 1). The microbiota in the dissimilar group appears to be clearly separated between the recipients and donors compared with that in the most similar group in the Principal Coordinate analysis plots for weighted Unifrac distance (Supplementary Fig. 2A). Using permutational analysis of variance (PERMANOVA), the dissimilar group showed the highest value for F-statistic (11.61) among three groups and statistical significances of dissimilarity between donor and recipient were found in the similar and dissimilar groups (*p* = 0.002 and 0.001, respectively) but not in the most similar group (*p* = 0.283). In the analysis of similarities (ANOSIM), similar results were also found (Supplementary Fig. 2B).

**Figure 2:**
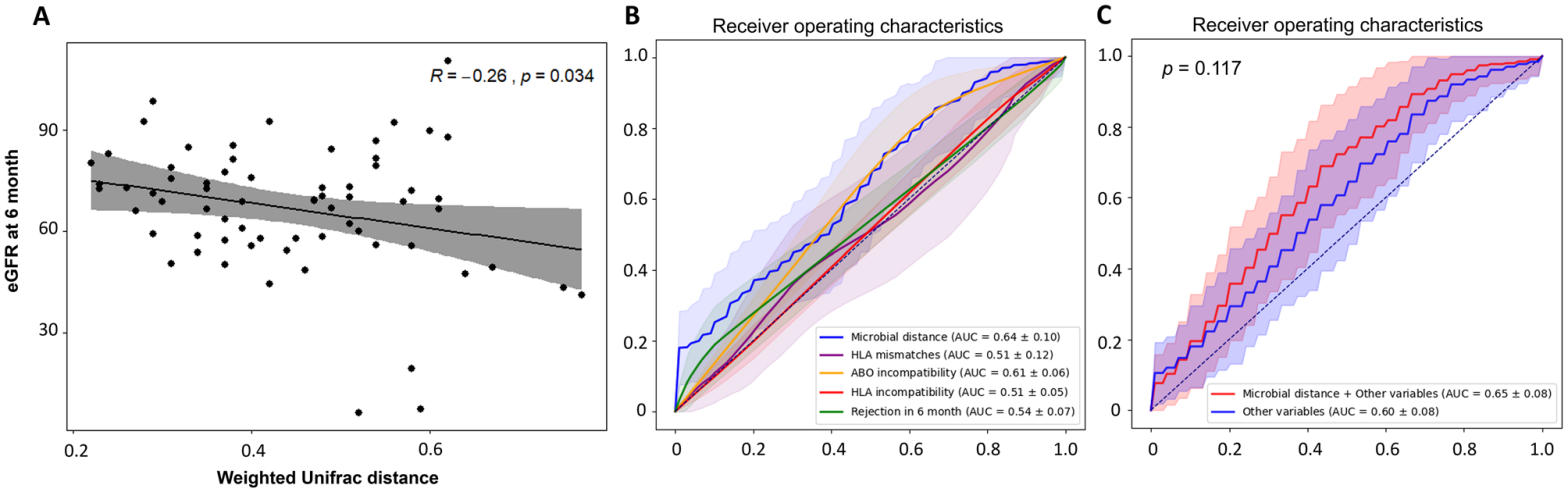
Association of the microbial distance with renal function at 6 months post-transplantation. (**A**) Linear regression analysis of the correlation between the estimated glomerular filtration rate (eGFR) at 6 months post-transplantation and weighted UniFrac distance of the microbiome. Solid line, regression line; shaded areas, 95% confidence interval. (**B**) Receiver operating characteristic curves for allograft dysfunction. The colored area represents 95% confidence interval of ROC curves. Blue line, microbial distance; orange line, number of HLA mismatches; red line, HLA incompatibility; green line, rejection in 6 months. (**C**) Receiver operating characteristic curves for allograft dysfunction according to clinical variables with (red) and without (blue) microbial distance. The colored area represents 95% confidence interval of ROC curves. Red line, microbial distance with clinical variables including recipient’s age, sex, body mass index, hypertension, diabetes, HLA/ABO compatibility, HLA mismatches and donor-recipient relationship; blue line, clinical variables including recipient’s age, sex, body mass index, hypertension, diabetes, HLA/ABO compatibility, HLA mismatches and donor-recipient relationship.

In comparing the presence or absence of specific OTUs according to distance groups, neither donors nor recipients showed a significant difference in microbial families among distance groups (Supplementary Fig. 3A). Conversely, in comparing the relative abundance of each OTU according to distance groups, we found significant differences of the relative abundance of the families *Bacteroidaceae, Prevotellaceae,* and *Enterobacteriaceae* among distance groups for donors but not for recipients (*q* = 0.030, 0.034, and 0.030, respectively. Supplementary Fig. 3B). The closer the distance of microbiota in donors, *Bacteriodaceae* and *Enterobacteriaceae* were increased while *Prevotellaceae* was decreased.

**Figure 3:**
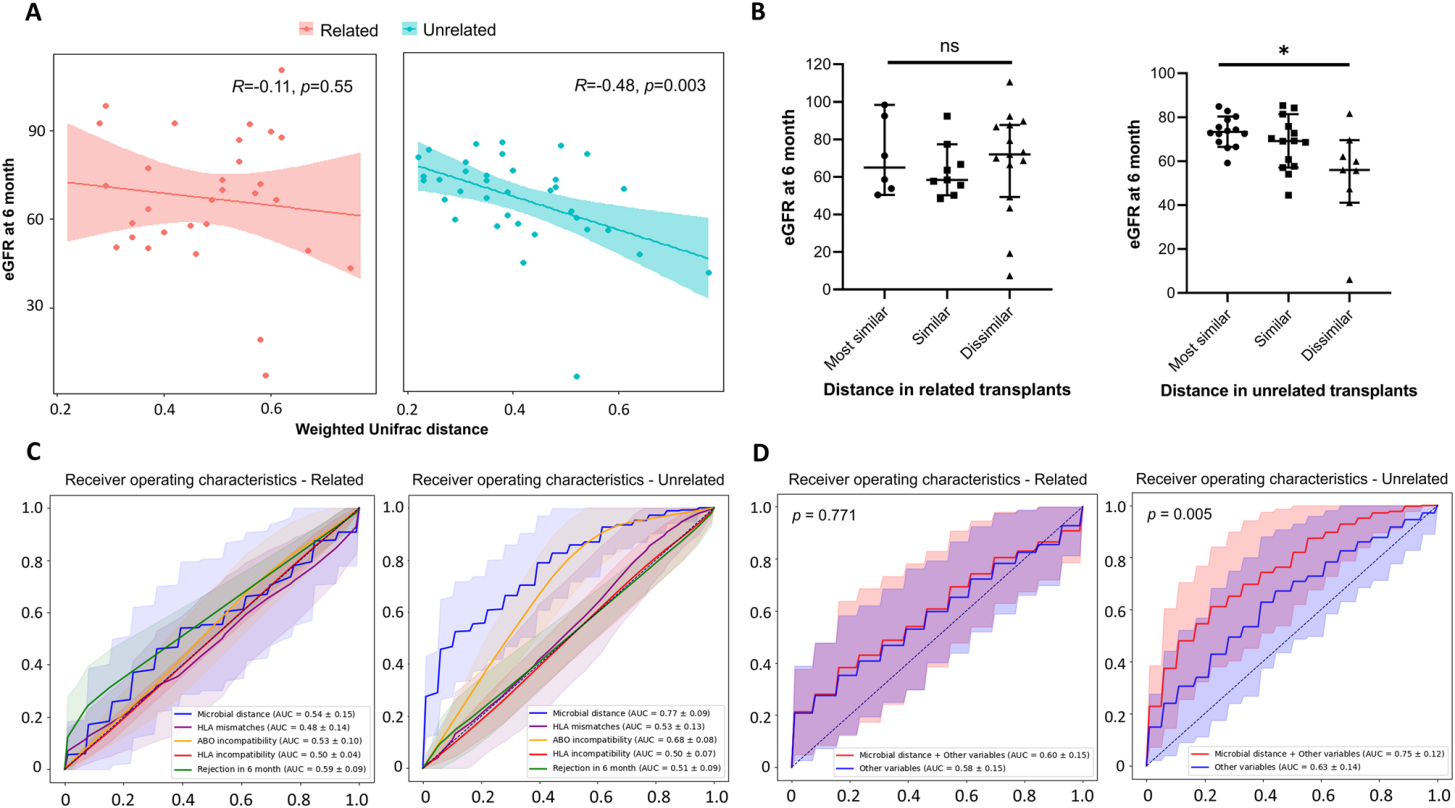
Difference of association between microbial distance and allograft function after 6-month transplantation according to relationship of donor and recipient. (**A**) Linear regression analysis of the correlation between the eGFR at 6 months post-transplantation and the weighted UniFrac distance of the microbiome in each subgroup of related and unrelated transplants. Solid line, regression line; shaded areas, 95% confidence interval. (**B**) Comparison of eGFR at 6 months post-transplantation between the distance groups in related transplants (left) and unrelated transplants (right). ns, not significant; * p < 0.05. (**C**) Receiver operating characteristic curves for allograft dysfunction in related pairs (left) and unrelated pairs (right). The colored area represents 95% confidence interval of ROC curves. Blue line, microbial distance; orange line, number of HLA mismatches; red line, HLA incompatibility; green line, rejection at 6 months. (**D**) Receiver operating characteristic curves for allograft dysfunction in related (left) and unrelated pairs (right) according to clinical variables with (red) and without (blue) microbial distance. The colored area represents 95% confidence interval of ROC curves. Red line, microbial distance with clinical variables including recipient’s age, sex, body mass index, hypertension, diabetes, HLA/ABO compatibility, HLA mismatches and donor-recipient relationship; blue line, clinical variables including recipient’s age, sex, body mass index, hypertension, diabetes, HLA/ABO compatibility, HLA mismatches, and donor-recipient relationship.

### Early allograft function and the microbial distance between transplant pairs

The correlation between the estimated glomerular filtration rate (eGFR) and microbial distance at 6 months after transplantation was assessed to evaluate the clinical effect of microbial distance in the early post-transplant phase. Although there were no statistical differences in eGFR between the three microbial distance groups (*p* = 0.205), interestingly, we found that the eGFR at 6 months after transplant showed a negative linear correlation with the microbial distance (*p* = 0.034, Fig. 2A). In addition, a negative correlation between microbial distance and eGFR was maintained even after adjustment for clinical factors, including the recipients’/donors’ age and gender, recipients’ body mass index, HLA mismatch numbers, ABO- and HLA- compatibility, diabetes mellitus, and the relationship between the donor and recipient (*p* = 0.016).

Subsequently, we explored whether microbial distance could predict allograft dysfunction at 6 months after kidney transplantation. We defined allograft dysfunction as eGFR < 60 mL/min/1.73 m^2^ at 6 months post-transplantation; the receiver operating characteristic (ROC) for allograft dysfunction was compared between microbial distance and other immunologic factors. Overall, the area under ROC curve (AUROC) of microbial distance was similar to those of other immunologic variables such as HLA-incompatibility, ABO-incompatibility, the number of HLA mismatches, and acute rejection episodes within the first 6-months after transplantation (Fig. 2B). Furthermore, microbial distance did not additively elevate the predictability of 6-month allograft function (*p* = 0.117, Fig. 2C).

### Effect of microbial distance on early allograft function according to donor-recipient relationship

As we found a significant difference in the microbial distance according to the donor–recipient relationship in terms of the baseline characteristics, we performed subgroup analysis between 30 related and 37 unrelated pairs. Unrelated pairs had a higher proportion of male recipients and a higher number of HLA mismatches than related pairs (Supplementary Table 2). There was no significant difference in the eGFR at 6 months after transplantation between related and unrelated pairs (67.0 ± 22.5 mL/min/1.73 m^2^ and 66.5 ± 15.3 mL/min/1.73 m^2^, respectively; *p* = 0.985). In the linear regression analysis stratified by related and unrelated pairs, a significant correlation between the microbial distance and 6-month allograft function was found only in unrelated pairs (R = − 0.48, *p* = 0.003), and the correlation was not significant in the related pairs (R = − 0.11, *p* = 0.550; Fig. 3A). Furthermore, in the categorical comparison of distance, the dissimilar group showed a significantly lower eGFR at 6 months than the most similar group only in the unrelated pairs, not in the related pairs (*q* = 0.012, Fig. 3B).

When we compared the predictability of allograft dysfunction of microbial distance and other variables in related and unrelated pairs, microbial distance not only showed significantly higher AUROC compared to immunologic factors except ABO incompatibility in unrelated pairs (*p* = 0.18, 0.011, 0.016, and 0.027 compared to ABO incompatibility, HLA incompatibility, HLA mismatch number, and rejection in 6 month, respectively. Figure 3C) but also showed significantly elevated predictability combined with clinical variables compared to it of clinical variables alone (*p* = 0.005, Fig. 3D).

### Different microbes between donor and recipient pairs according to microbial distance

**Figure 4:**
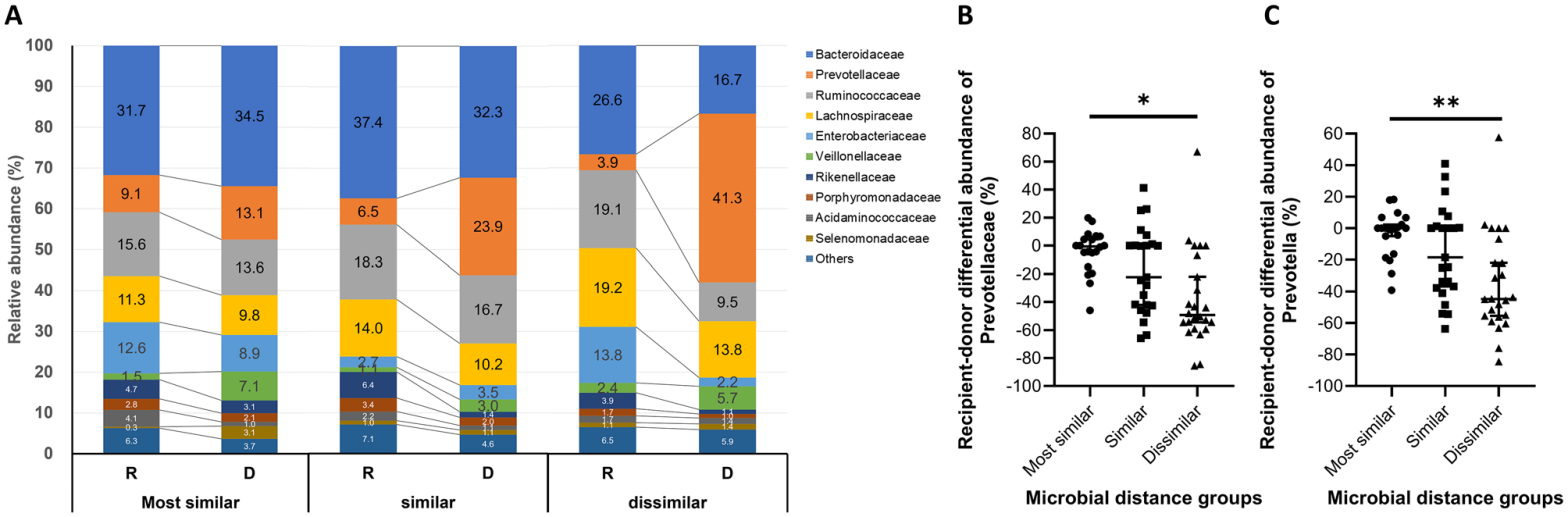
Differential abundance of microbiota between donor and recipient according to distance groups. (**A**) Relative abundance of major families between donor and recipient. (**B**) Comparison of differential abundance of family *Prevotellaceae* in three distance groups. *, *q* < 0.05. (**C**) Comparison of differential abundance of genus *Prevotella* in three distance groups. **, *q* < 0.01.

To find the members of microbiota causing difference of distance between donors and recipients, we compared the differential relative abundance of each OTU in donor-recipient pairs among three distance groups (Fig. 4A). Of the major families which have average relative abundance over 1% in this cohort, the differential abundance of *Prevotellaceae* between donor and recipient was significant among distance groups (*p* < 0.001, *q* = 0.006, Fig. 4B). Of the major genera, only the differential abundance of *Prevotella*, which is in the family *Prevotellaceae*, showed a significant difference among distance groups (*p* < 0.001, *q* = 0.011, Fig. 4C). Dissimilar group showed a high relative abundance of *Prevotellaceae* and *Prevotella* in donors and a low relative abundance of *Prevotellaceae* and *Prevotella* in recipients. However, the differential abundance of *Prevotellaceae* or *Prevotella* alone showed no significant association with eGFR at 6 months post-transplantation.

### Adverse events according to the microbial distance

**Figure 5:**
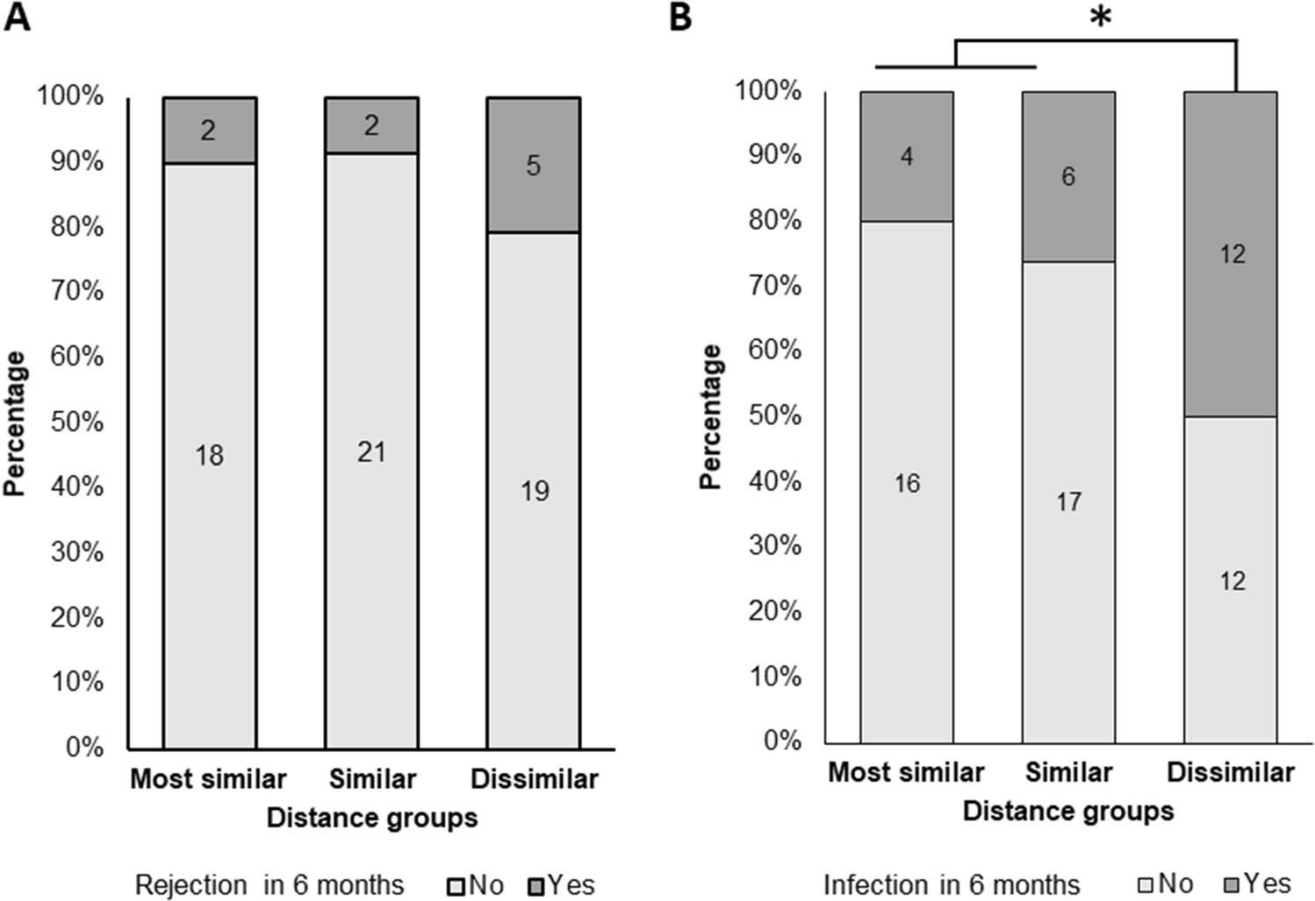
Incidence of rejection (**A**) and infection (**B**) within 6 months after transplantation according to the distance groups. * p < 0.05.

To explain how the microbial distance affects kidney function in the early post-transplant period, we identified episodes of rejection and infection within 6 months in each distance group. The episodes of acute rejection occurred in 2/20 (10.0%) pairs in the most similar group and 5/24 (20.8%) in the dissimilar group, which was approximately two times higher than that of the most similar group (Fig. 5A). However, the difference was not statistically significant among the groups according to the chi-square test. Episodes of infection occurred in 4/20 pairs (20.0%) in the most similar group, 6/23 (26.1%) in the similar group, and 12/24 (50.0%) in the dissimilar group. A comparison of the most similar and similar groups with dissimilar group revealed that dissimilar group had a significantly higher incidence of episodes of infection (*p* = 0.025) (Fig. 5B). In the subgroup analysis of infection according to related and unrelated pairs, there were more episodes of infection in the unrelated pairs; however, this finding was not statistically significant (14.3% in the most similar, 28.6% in similar, and 55.6% in dissimilar groups; *p* = 0.186).

### Changes of recipients’ microbiota after transplantation according to distance groups

Among the 67 transplant pairs, the recipients of 47 pairs had follow-up stool samples after transplantation. In these recipients, we additionally analyzed the changes of microbiota from pretransplant samples to post-transplant samples according to distance groups. However, the changes of microbiota was not statistically different according to microbial distance group (Supplementary Fig. 4).

## Discussion

Immunologic barriers, such as anti-HLA donor-specific antibodies or anti-ABO blood group antibodies, have been a major problem for successful kidney transplantation. Despite recent advances in immunomodulatory therapies and monitoring systems, there are many unresolved problems. Microbiota may represent one of the important parts of immune modulation in transplantation patients. In this study, a negative correlation was observed between early allograft function and the microbial distance of donor–recipient pairs, especially in unrelated pairs rather than in genetically related pairs. Interestingly, the microbial distance showed a more significant association with decline in eGFR than other genetic and immunologic factors, such as the number of HLA mismatches and HLA incompatibility in unrelated pairs. Our results suggest an important role of microbiota in maintenance of allograft function.

Previously, several studies revealed comparable transplant outcomes between non-relative and relative donors^3,4^. However, explanations for comparable graft outcomes that overcome genetic differences between unrelated and related transplants are limited. Similar to close relationships between related donors and recipients, which largely determine donation decisions in unrelated transplants, some unrelated donor–recipient pairs might share a similar environment with high interaction in their daily life. As expected, in our study, the unrelated pairs showed closer microbial distances than related pairs, and they more frequently shared meals, reflecting an intimacy between these unrelated donors and recipients. Because humans shed approximately 30 million bacterial cells per hour, sharing a common space has been shown to affect an individual’s microbiota composition^9–11,17,18^. Furthermore, as dietary habits directly affect the intestinal microflora^19^, sharing spaces and dietary contents could affect the UniFrac distance, which represents similarity between microbial communities by relative abundance of each taxon. As spousal donors are major candidates for living unrelated donors in most organ transplant centres, most of the unrelated donors in our study were spousal donors. In this study, spousal donors reported a significantly higher frequency of sharing meals (Supplementary Fig. 1B) and had a particularly closer microbial distance (Supplementary Fig. 5) than related donors. These results suggest that married couples have similar microbial communities because they eat together and share their living environment. The similarity of these microbial communities can be suggested as an important factor that overcomes the disadvantages of spousal donors who have genetic disparity with higher numbers of HLA mismatch than living related donors, and spousal donors have comparable outcomes with living related donors^3,4,20^.

Although the similarity in microbiota due to sharing of environments is well known^10,11^, its biological meaning, such as an impact on the immune system, remains unclear. Previous studies reported that, at homeostasis, commensal and food antigens are presented to T-cells, leading to their differentiation to the commensal-specific T-regulatory cells^21,22^. These T-regulatory cells regulate commensal-specific responses and are part of the mucosal firewall^21,23^. When allografts have a very dissimilar microbial community before transplantation, new commensal antigens from donor may activate the inflammatory milieu with changes in the T-cell subsets of recipients. In an animal study, a subset of CD8 T-cells of mice in pet stores with easier contacts with human showed recapitulated aspects of human CD8 T-cell differentiation and distribution, unlike feral or laboratory mice^24^.

In the present study, the members of microbiota that were the most differential in the three distance groups were family *Prevotellaceae* and genus *Prevotella*. The role of *Prevotella* within gut microbiota and their effects on the host are poorly understood, and conflicting interpretations have been reported. In some studies, *Prevotella* was related to increased short chain fatty acid production, which induces a tolerogenic and anti-inflammatory profile of T cells^25–28^. In other studies, *Prevotella* was related to an increased susceptibility to inflammatory disease such as rheumatoid arthritis^29,30^ and has been reported to increase mucosal Th17 immune response and neutrophil recruitment^31,32^. Nevertheless, *Prevotella* may have a significant role in regulating the immune system, including T cell subsets. In our study, recipients in the dissimilar distance group showed a lower proportion of *Prevotellaceae* compared to donors in the same group as well as recipients in the other two groups. Our results suggest that differential immunologic responses caused by the differences of gut microbiota between donor and recipient , and *Prevotella* in the dissimilar distance group may affect early allograft dysfunction after kidney transplantation.

Furthermore, although the statistical significance was not sufficient due to the small numbers of pairs, the increased incidence of both rejection and infection in the dissimilar group can be considered as another evidence of the alteration of the immune system in recipients. Therefore, although the microbial composition similarity caused by cohabitation was estimated to contribute to the similar immune profiles or specificity between donor and recipient, whether or not it directly affects kidney transplantation outcomes should be clarified further.

The interest in the role of microbiota in modulating transplant outcomes is increasing. Oh et al. showed differences in the total intestinal microbiota of transplant recipients with or without rejection after small bowel transplantation^15^. Fricke et al. reported that oral and rectal microbiota were associated with transplant rejection and that rectal microbiota was associated with infection in kidney transplant recipients^33^. Moreover, Lee et al. found that post-transplant diarrhoea was associated with gut dysbiosis^34^. Our study provides further information about the role of microbiota in transplant outcomes and suggests the need for an approach to improve transplant outcomes by controlling donors’ as well as recipients’ microbiota in transplant pairs.

The main limitation of our study is that the mechanism of interaction between microbial similarity and graft function was not validated. The hypothesis about the mechanisms related to infection and immune alteration suggested by the clinical results in this study needs to be verified in future experimental studies. Another limitation of our study is the small number of samples. In order to maintain statistical significance with the small number of pairs, the distance group could not be further divided. In addition, it is suspected that the statistical significance of the infection events according to the distance group cannot be achieved due to the limitation of the number of pairs. Therefore, large-scale research is needed to overcome this limitation.

In summary, our results suggest that the distance between the donor–recipient microbial community before transplantation affects early allograft function and that it might be associated with increased infection incidence within 6 months after transplantation. In addition, this association between graft function and microbial distance was more pronounced in unrelated donors than in related donors. Furthermore, environmental factors might play an important role in transplants, especially in genetically unrelated donor transplants. Our study elucidates the importance of the microbial community, which might be one of the unknown environmental factors and traditional genetic factors that should be considered in the management of recipients after transplantation.

## Materials and methods

### Study participants

This study was approved by the Medical Ethics Committee of the Seoul National University Hospital (institutional review board [IRB] number: 1703-062-839) and complied with the Declaration of Helsinki. This study included only participants who provided informed consent and agreed to provide stool samples for deposition in a human stool repository (IRB number: 1802-062-921). The human stool repository included stool samples from kidney transplant donors and recipients collected before transplantation and stool samples from recipients collected after transplantation (IRB number: 1703-062-839). We enrolled pairs of donors and recipients registered in the human stool repository, where the pretransplant stool samples of both donors and recipients are available.

### Clinical information of recipients and donors

We compiled demographic information, including age, sex, and body mass indices of the kidney recipients, and identified whether the recipients had comorbidities, including hypertension and DM, based on their clinical and medication history in the electronic medical record system. The causes of ESRD, which was presumed clinically or pathologically, were also identified. We obtained ABO incompatibility, HLA incompatibility, and number of HLA mismatches, as they could be important factors related to post-transplant outcomes. The relationship between donors and recipients was classified as related and unrelated including spouses and other relationships. In addition, information about the donor’s age, sex, and body mass indices was reviewed.

### Stool DNA extraction and MiSeq sequencing

Participants were asked to store samples below 0 °C in their refrigerator and carry the samples to our centre in stainless steel cooler box within 24 h of collection. Upon arrival, the stool samples were immediately stored in a deep freezer at − 80 °C and processed within 3 months of collection. DNA extraction from the stool samples was performed using the QIAamp Fast DNA Stool Mini Kit (Qiagen, Hilden, Germany), according to the manufacturer’s instructions^35^. The V4-5 variable regions of the 16S rRNA gene was amplified using extracted DNA. The amplification was performed according to the protocol for preparing a 16S metagenomics sequencing library with the MiSeq system (Illumina, Inc., San Diego, CA, USA). The amplicons of each sample were purified using Agencourt AMPure XP beads (Beckman Coulter, Indianapolis, IN, USA), and the purified amplicons were quantified using a PicoGreen dsDNA Assay Kit (Invitrogen, Carlsbad, CA, USA). The equimolar concentration of each library was pooled and sequenced in the Illumina MiSeq system (250-bp paired ends), according to the manufacturer’s instructions. The obtained sequences in this study are available through the European Nucleotide Archive at the European Bioinformatics Institute under accession number PRJEB36456.

### Sequence data analysis including calculation of the microbial distance

For microbiota analysis, the obtained sequence reads were analysed using the CLC genomic workbench v.11.0.1 with the Microbial Genomics Module (Qiagen). Briefly, raw sequences were merged, and sequences with low-quality scores or short read lengths (< 400 bp) and chimeric reads were removed using the USEARCH pipeline v.11.0.667 (https://www.drive5.com/usearch). Primer sequences were removed from the merged sequences, and clustered into operational taxonomic units (OTUs) based on 97% sequence identity; then, taxonomic positions of representative sequences in each OTU were assigned against the EzTaxon-e reference database^36^. Sequence read numbers were normalized by random subsampling for α‐diversity indices by Mothur^37^. Principal coordinate analysis (PCoA) based on the weighted UniFrac distance was carried out to compare the microbiota composition among samples using Calypso^38^. To quantify the microbial distance, we used beta-diversity metrics between samples of donors and recipients based on the weighted UniFrac distance^39,40^.

The weighted UniFrac distance is a distance metric used to compare microbial communities by calculating the distance according to the branch of the phylogenetic tree, considering the abundance of organisms^41,42^. To obtain weighted UniFrac distances, the 16 s DNA sequences were aligned using the MUSCLE tool of the CLC Microbial Genomics Module, and a phylogenetic tree was constructed using the Maximum Likelihood method of the CLC genomic workbench. Because there is no absolute value for the degree of microbial composition similarity, we defined their “relative” microbial similarity as follows. After calculating the weighted UniFrac distances, we divided the study subjects into three groups according to the tertiles of weighted UniFrac distances as most similar, similar, and dissimilar groups. Thereafter, we compared their baseline characteristics and outcomes according to the similarity groups.

### Clinical outcomes

To determine the effect of the similarity of microbiota on transplant outcomes, data about the recipients’ renal function, post-transplant acute rejection, and infection events were collected. The recipients’ renal function was measured by eGFR at 6 months after transplantation using the Chronic Kidney Disease Epidemiology Collaboration calculation formula^43^. Episodes of acute rejection included cell-mediated and antibody-mediated rejections identified based on the pathology report of the renal biopsy within 6 months after transplantation. In our centre, we performed protocol graft biopsies at 2 weeks after transplantation irrespective of whether there was evidence of an allograft injury and performed indication graft biopsies at any time if clinically suspected for rejection. In the present study, we only included episodes of acute rejection diagnosed by indication graft biopsies. Infection events were defined when viral or bacterial pathogens were identified by culture or when viral DNA was detected by polymerase chain reaction in any specimen (e.g., urine, blood, and bronchial aspiration) within 6 months after transplantation.

### Statistical analysis

For the baseline characteristics, the continuous variables are expressed as a mean and standard deviation, and the categorical variables are expressed as a percentage. Differences in clinical characteristics between groups were evaluated using the Mann–Whitney U and Kruskal–Wallis tests. PERMANOVA and ANOSIM, performed using the vegan package in R, were used to determine whether the distance between donor and recipient in three microbial distance groups are statistically different. The correlation between the weighted UniFrac distance and eGFR was analysed by linear regression. The receiver operating characteristic (ROC) analysis was used to compare predictive models, and the AUROC was calculated with a 95% confidence interval (CI) achieved by the bootstrapping method. For simple comparison of baseline characteristics and linear regression analysis, *p* values less than 0.05 were considered significant. For multiple comparisons after Kruskal–Wallis test with multiple variables, false discovery rate was applied for multiple testing, and *q* values of less than 0.05 were considered significant. Permutation tests were performed to determine *p* values between ROC analyses. The statistical analyses were performed using R version 3.5.0 (R Core Team, Vienna, Austria), Stata version 15.1 (StataCorp), Python 3.7 (Python Software Foundation, https://www.python.org/) and GraphPad Prism version 8.1.1 (GraphPad Software, San Diego, California, USA).

## Supplementary information

Supplementary Table 1.

Supplementary Table 2.

Supplementary Figures.

## Data Availability

The obtained sequences in this study are available through the European Nucleotide Archive (ENA) at the European Bioinformatics Institute (EBI) under Accession Number PRJEB36456.

## Abbreviations

AUROC: Area under the receiver operating characteristic curve
CI: Confidence interval
DM: Diabetes mellitus
eGFR: Estimated glomerular filtration rate
ESRD: End-stage renal disease
HLA: Human leukocyte antigen
IRB: Institutional review board
OTUs: Operational taxonomic units
PCoA: Principal coordinate analysis
ROC: Receiver operating characteristic
