# Supplementary material for: Effect of the similarity of gut microbiota composition between donor and recipient on graft function after living donor kidney transplantation
Source: Sci Rep. 2020 Nov 3;10:18881. doi: 10.1038/s41598-020-76072-8 (PMC7641223; doi:10.1038/s41598-020-76072-8)
Supplement: Supplementary file 2 — Supplementary Table 2. [file 41598_2020_76072_MOESM2_ESM.docx]

Supplementary table 2. Baseline characteristics of transplant pairs according to their relationship.

|  | Related | Unrelated |  |
| --- | --- | --- | --- |
| Variables | N=30 | N=37 | *P* |
| Recipient |  |  |  |
| Age (years) | 44.4±16.1 | 50.6±9.2 | 0.175 |
| Male gender (%) | 12 (40.0) | 26 (70.3) | 0.013 |
| BMI (kg/m^2^) | 22.9±4.9 | 22.9±3.2 | 0.93 |
| DM (%) | 8 (26.7) | 12 (32.4) | 0.608 |
| HTN (%) | 19 (63.3) | 31 (83.8) | 0.056 |
| ABO incompatibility (%) | 6 (20.0) | 12 (32.4) | 0.254 |
| HLA incompatibility (%) | 2 (6.7) | 5 (13.5) | 0.362 |
| Number of HLA mismatch | 1.9±1.4 | 4.2±1.3 | <0.001 |
| Donor |  |  |  |
| Age (years) | 45.1±12.3 | 50.2±9.4 | 0.113 |
| Male gender (%) | 15 (50.0) | 12 (32.4) | 0.145 |
| **Weighted UniFrac distance** | **0.48±0.13** | **0.41±0.13** | **0.025** |
