# Supplementary material for: Effect of the similarity of gut microbiota composition between donor and recipient on graft function after living donor kidney transplantation
Source: Sci Rep. 2020 Nov 3;10:18881. doi: 10.1038/s41598-020-76072-8 (PMC7641223; doi:10.1038/s41598-020-76072-8)

**Effect of the similarity of gut microbiota composition between donor and recipient on graft function after living donor kidney transplantation**

Ji Eun Kim^1,2^, Hyo-Eun Kim^1^, Hyunjeong Cho^3^, Ji In Park^4^, Min-Jung Kwak^5^, Byung-Yong Kim^5^, Seung Hee Yang^6^, Jung Pyo Lee^7^, Dong Ki Kim^1,6^, Kwon Wook Joo^1,6^, Yon Su Kim^1,6^, Bong-Soo Kim^8^, and Hajeong Lee^1^*

^1^ Department of Internal Medicine, Seoul National University Hospital, Seoul

^2^ Department of Internal Medicine, Korea University Guro Hospital, Seoul

^3^ Department of Internal Medicine, Chungbuk National University Hospital, Cheongju

^4^ Department of Internal Medicine, Kangwon National University Hospital, Chuncheon

^5^ ChunLab, Inc., Seoul

^6^ Kidney Research Institute, Seoul National University, Seoul, Korea

^7^ Department of Internal Medicine, Seoul National University Boramae Hospital, Seoul

^8^ Department of Life Science, Multidisciplinary Genome Institute, Hallym University, Chuncheon, Republic of Korea

***Corresponding author:** Hajeong Lee, MD, PhD

Department of Internal Medicine, Seoul National University Hospital, 103 Daehakro, Jongno-gu, Seoul 03080, Korea

Tel: 82-2-2072-4905

E-mail: [mdhjlee@gmail.com](mailto:mdhjlee@gmail.com)

Supplementary figure 1. Comparison of number of sharing meals according to donor-recipient relationship. A shows the number of meals in unrelated and related transplant pairs. B shows the comparison between related transplant pairs and unrelated donor pairs divided into spousal donors and other donors. *, p < 0.05.


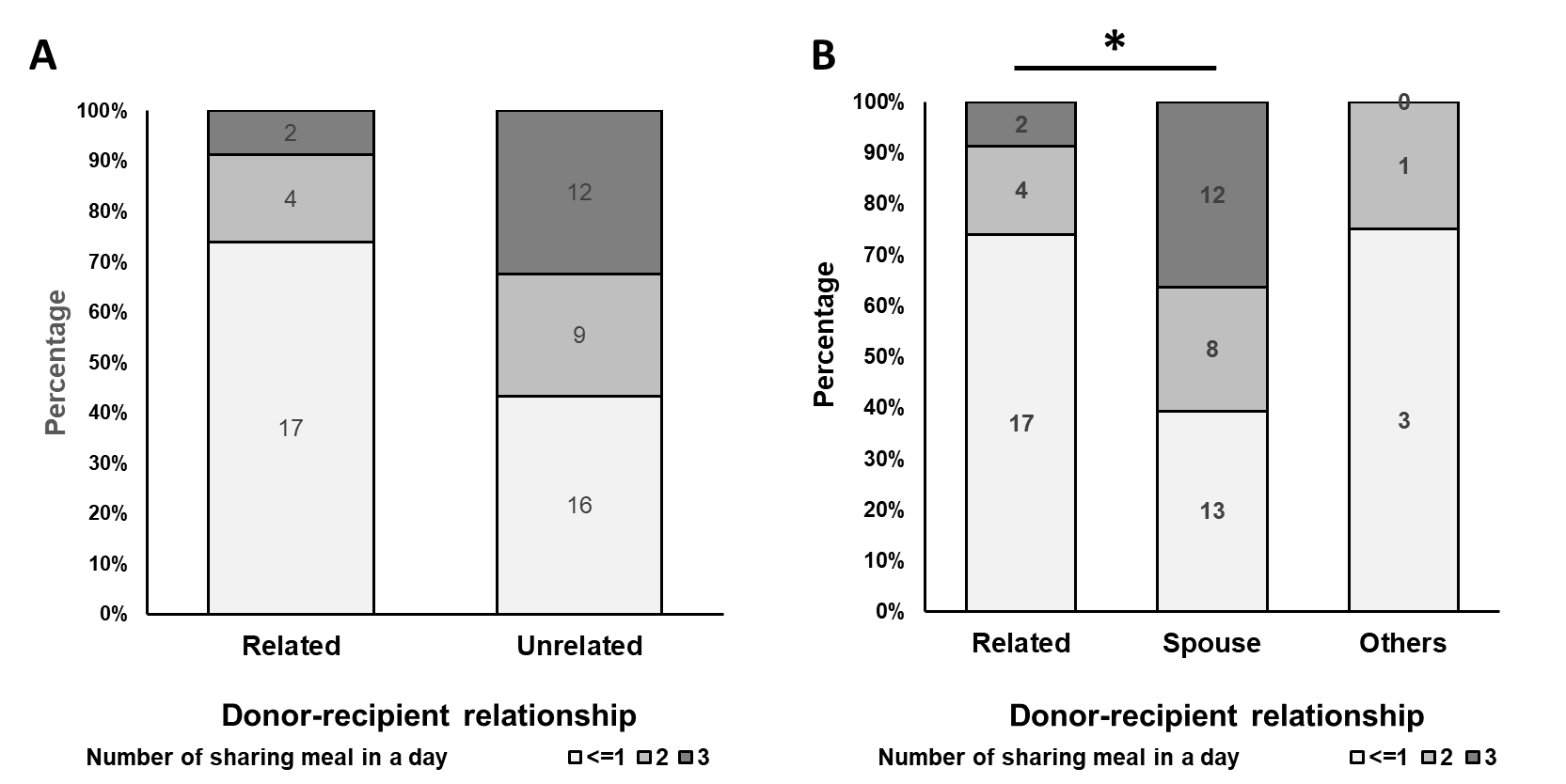


Supplementary figure 2. Beta diversity analysis among three groups. A. Principal coordinate analysis (PCoA) plots for weighted Unifrac distance. The first two axes of the PCoA are represented by principal coordinate axis 1 (PCoA1) and principal coordinate axis 2 (PCoA2). B. Analysis of similarities between and within groups. ‘Between groups’ shows dissimilarity scores between donors and recipients, while ‘Donor’ and ‘Recipient’ show dissimilarity scores within donors and recipients, respectively.


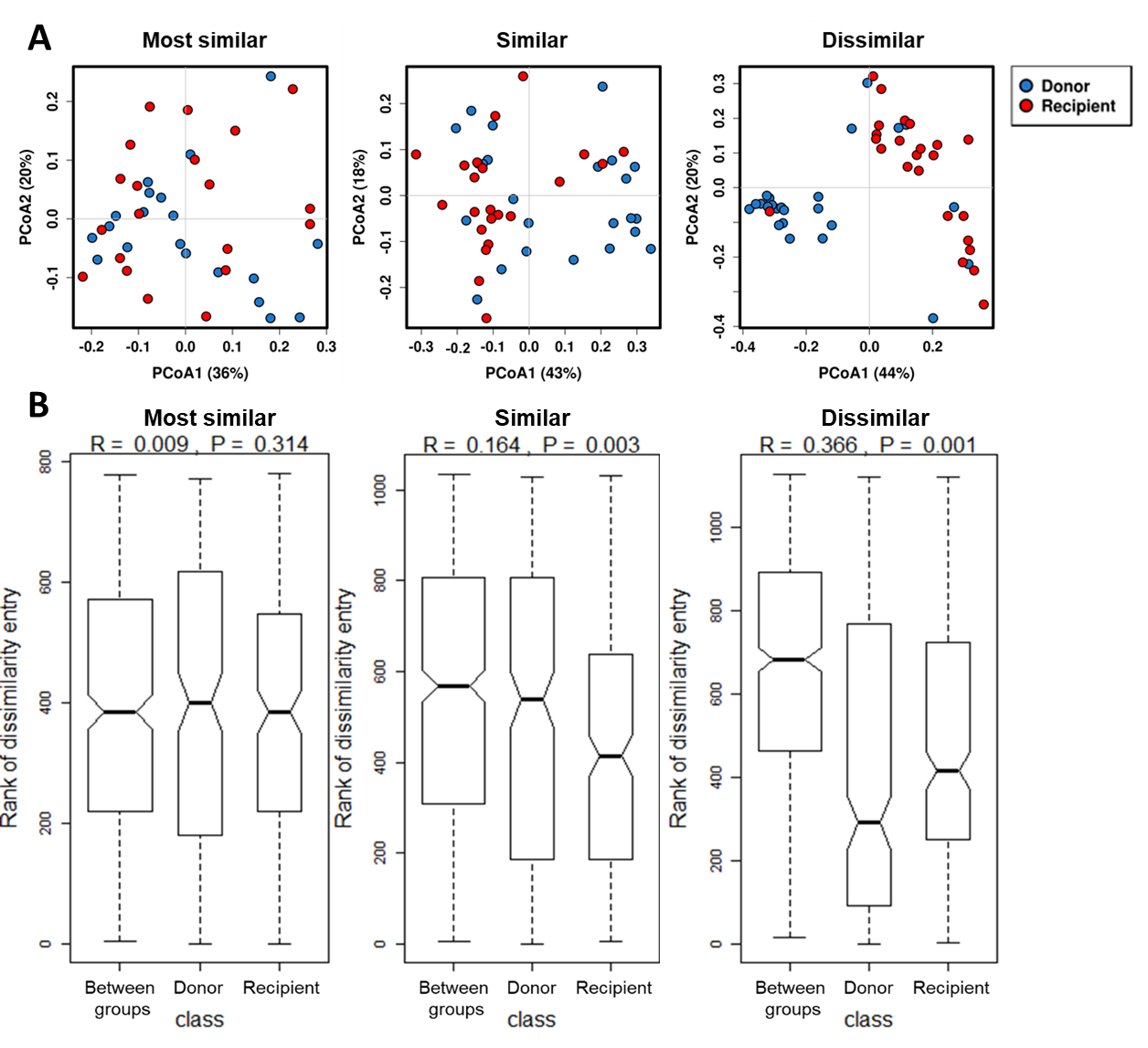


Supplementary figure 3. Major microbial families according to distance groups in recipients and donors. A. Presence percentages of each microbial families in groups. B. Logarithm-changed relative abundances of microbial families in groups. Major microbial families were defined as the families which have average relative abundance over 1% in the cohort.


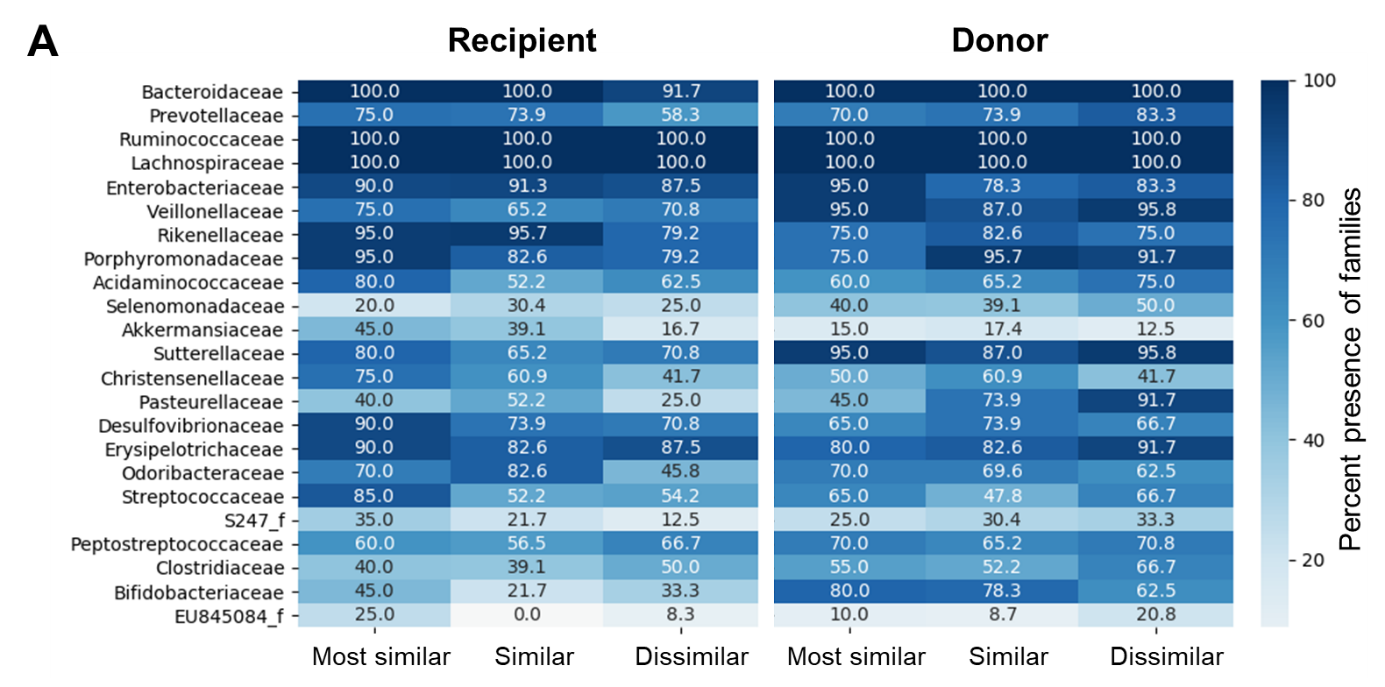


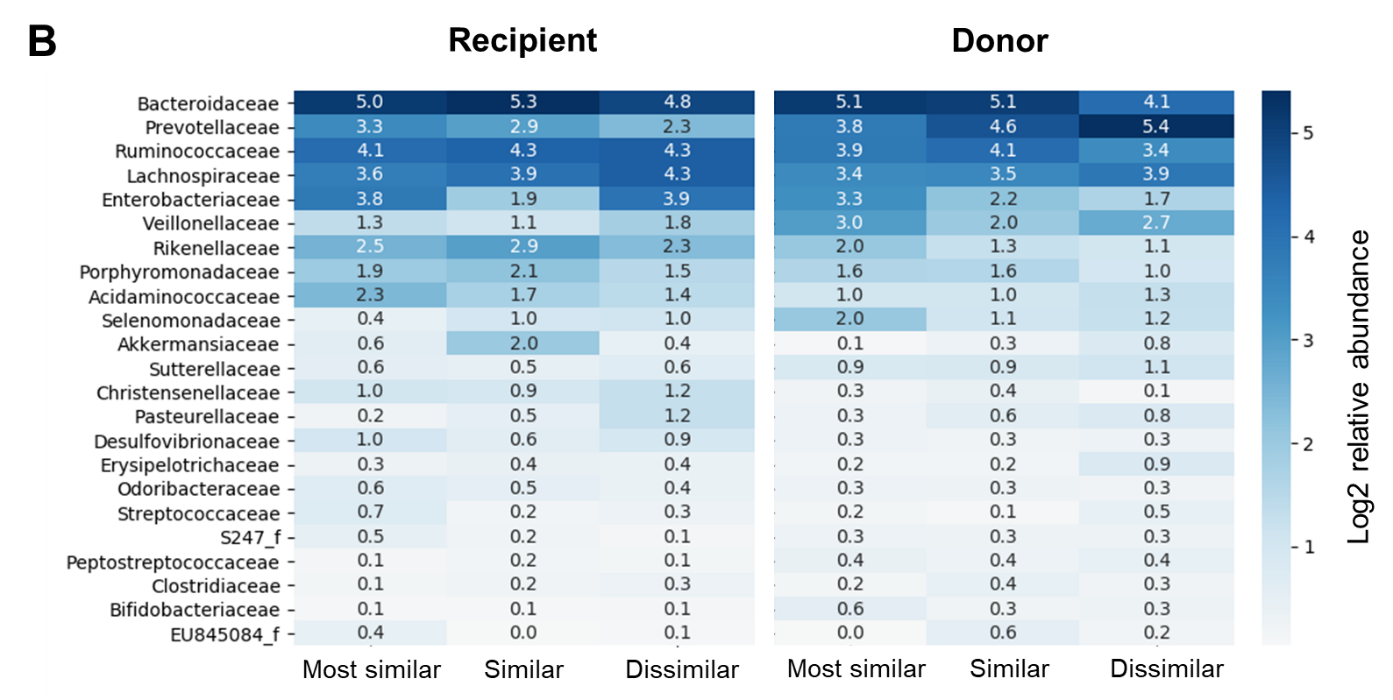


Supplementary Figure 4. Microbial distance in Related and Unrelated transplants including spousal and other transplants. *, *p* < 0.05.

Supplementary Figure 5. Pretransplant and posttransplant relative abundances of major microbial families in distance groups of recipients. Posttransplant samples were collected at 3 months after kidney transplantation. Relative abundances are displayed as logarithmic scale. Pre, pretransplant; Post, posttransplant.


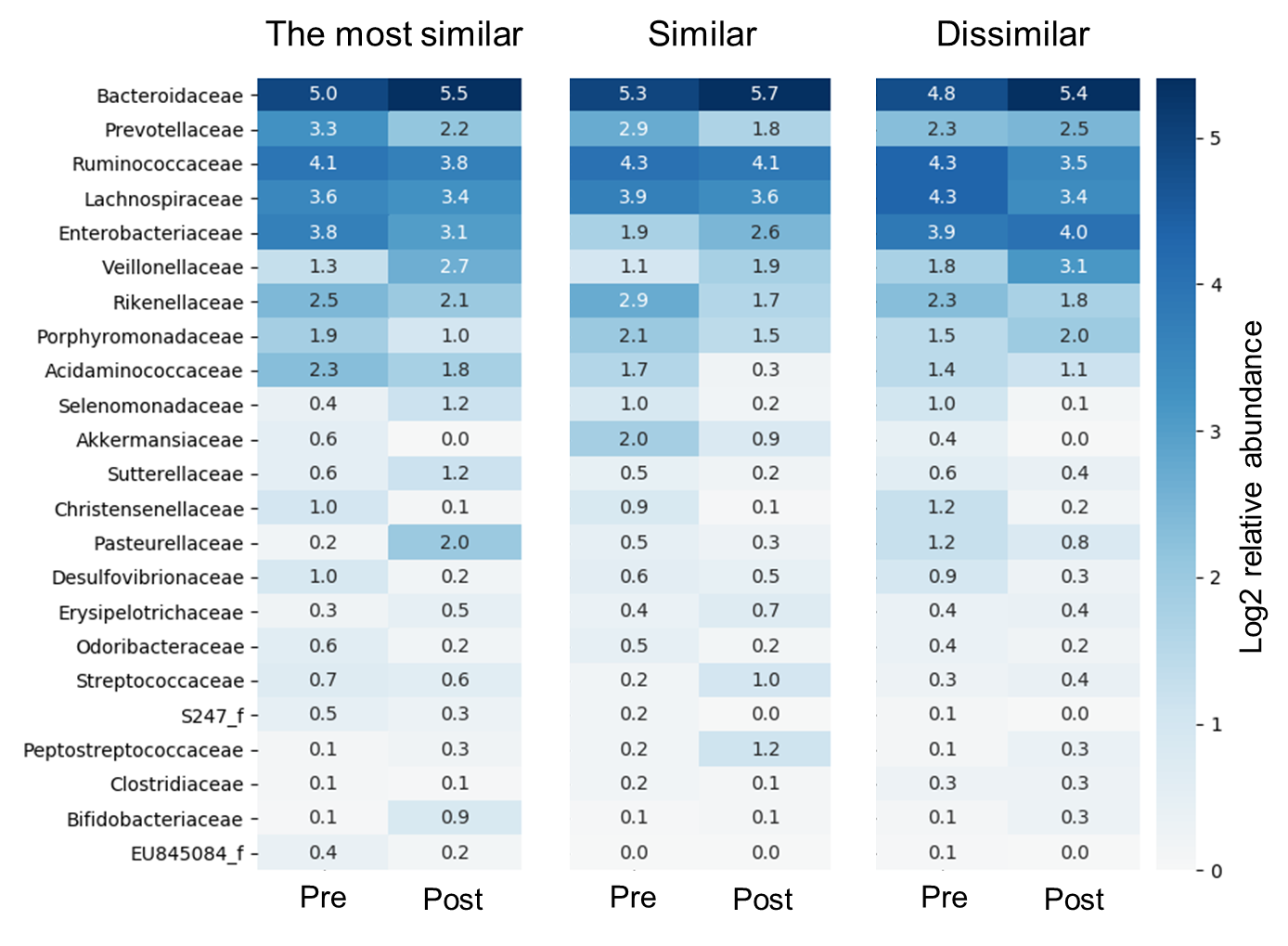

Supplement: Supplementary file 3 — Supplementary Figures. [file 41598_2020_76072_MOESM3_ESM.docx]
